# ALDH1 expression predicts progression of premalignant lesions to cancer in Type I endometrial carcinomas

**DOI:** 10.1038/s41598-021-90570-3

**Published:** 2021-06-07

**Authors:** Vei Mah, Yahya Elshimali, Alison Chu, Neda A. Moatamed, Jamar P. Uzzell, Jessica Tsui, Stephen Schettler, Hania Shakeri, Madhuri Wadehra

**Affiliations:** 1 4525 MacDonald Research Laboratories, Department of Pathology and Laboratory Medicine, David Geffen School of Medicine at UCLA, Los Angeles, CA 90095 USA; 2 Department of Pediatrics, David Geffen School of Medicine at UCLA, Los Angeles, USA; 3 Division of Cancer Research and Training, Department of Medicine, Charles R. Drew University of Medicine and Science, Los Angeles, USA; 4 Jonsson Comprehensive Cancer Center, David Geffen School of Medicine at UCLA, Los Angeles, USA

**Keywords:** Stem cells, Molecular medicine, Oncology

## Abstract

In type 1 endometrial cancer, unopposed estrogen stimulation is thought to lead to endometrial hyperplasia which precedes malignant progression. Recent data from our group and others suggest that ALDH activity mediates stemness in endometrial cancer, but while aldehyde dehydrogenase 1 (ALDH1) has been suggested as a putative cancer stem cell marker in several cancer types, its clinical and prognostic value in endometrial cancer remains debated. The aim of this study was to investigate the clinical value of ALDH1 expression in endometrial hyperplasia and to determine its ability to predict progression to endometrial cancer. Interrogation of the TCGA database revealed upregulation of several isoforms in endometrial cancer, of which the ALDH1 isoforms collectively constituted the largest group. To translate its expression, a tissue microarray was previously constructed which contained a wide sampling of benign and malignant endometrial samples. The array contained a metachronous cohort of samples from individuals who either developed or did not develop endometrial cancer. Immunohistochemical staining was used to determine the intensity and frequency of ALDH1 expression. While benign proliferative and secretory endometrium showed very low levels of ALDH1, slightly higher expression was observed within the stratum basalis. In disease progression, cytoplasmic ALDH1 expression showed a step-wise increase between endometrial hyperplasia, atypical hyperplasia, and endometrial cancer. ALDH1 was also shown to be an early predictor of EC development, suggesting that it can serve as an independent prognostic indicator of patients with endometrial hyperplasia with or without atypia who would progress to cancer (*p* = 0.012).

## Introduction

Endometrial carcinoma is the fourth most common cancer in women in the United States, and the most common gynecologic cancer in developed nations^1^. These carcinomas have been broadly classified into two types: Type 1 (endometrioid and mucinous carcinoma), which encompasses the large majority of endometrial cancers (~ 80%), and Type 2, which includes less common serous, clear cell, undifferentiated carcinoma and carcinosarcoma-types. Type 1 endometrial cancer is associated with long term unopposed estrogen stimulation, which is thought to contribute to endometrial hyperplasia, while type 2 is generally considered to be estrogen independent.

Endometrial hyperplasia is a pathologic condition defined as hyperplastic changes that occur in the endometrial glandular epithelium resulting in increased glandular proliferation in different shapes and irregular sizes, with an associated increase in gland to stroma ratio. While less than 2% of endometrial hyperplasia cases without cytological atypia progress to endometrial cancer, hyperplasia accompanied by atypia presents a significant clinical concern as it serves as a precursor to endometrial cancer^2^. In these cases, ~ 20–25% of patients will progress to malignancy, while in others, endometrial hyperplasia may regress without incident or detection^3^. Therefore, there is a need for better and more accurate biomarkers to diagnose and predict which precancerous lesions of the endometrium to treat.

Endometrial cancers consist of heterogenous cell populations that may derive from a single clone. The cellular heterogeneity is thought to occur from different somatic mutations and/or result from a renewable small subpopulation of cells, referred to as cancer stem cells (CSC), that have the capability of transforming from an epithelial to a mesenchymal phenotype^4^. Aldehyde dehydrogenase 1 (ALDH1) has been identified as a putative CSC marker in several cancer types^5^ and is expressed in endometrial cancer^6, 7^. While the body of evidence for ALDH1 expression having clinicopathological and prognostic value in a number of cancers grows, including colorectal cancer^8, 9^, bladder and prostate cancer^10, 11^, and breast cancer^12, 13^, less is known about its ability to predict the progression of pre-cancerous lesions to cancer.

Therefore, the aim of this study is to investigate the clinical utility of ALDH1 expression in pre-cancerous endometrial lesions. We test the hypothesis that ALDH1 expression can predict progression of pre-cancerous endometrial lesions to cancer.

## Materials and methods

### TCGA analysis

The expression of all 17 ALDH isoforms was analyzed through the CBioPortal for Cancer Genomics (http://cbioportal.org)^14^. 526 cases of uterus corpus endometrial carcinoma (PanCancer Atlas) were evaluated for EMP2 expression (mRNA expression z-scores relative to diploid samples). Data is summarized as an OncoPrint for multiple genes across a set of tumor samples (columns)^14^ as well as survival data dividing groups into altered versus unaltered mRNA.

### Cell culture

HEC1A and HEC1B cells (ATCC, Manassas, VA, USA) were cultured in McCoys or DMEM media supplemented with 10% fetal bovine serum, 1% l-glutamine, 1% sodium pyruvate and 1% penicillin–streptomycin at 37 °C in a humidified 5% CO_2_. Experiments were performed on cell lines within 3 months after resuscitation of frozen aliquots and were authenticated based on viability, recovery, growth, and morphology. Cell lines were tested monthly for mycoplasma (Lonza, Walkersville, MD, USA).

### Flow sort and cell proliferation

Semi-confluent cells were harvested using a 0.5% EDTA solution in HBSS. ALDH^high^ and ALDH^low^ subsets were isolated using an ARIA III flow cytometric machine (BD Biosciences) using the ALDEFLUOR assay kit (StemCell Technologies, Vancouver, BC) according the manufacturer’s guidelines. Briefly, 1 × 10^6^ cells were incubated in ALDEFLUOR assay buffer containing ALDH substrate or under identical conditions with 50 mmol/L of diethylaminobenzaldehyde, an ALDH inhibitor, as a negative control.

In order to compare the rate of proliferation between ALDH^high^ versus ALDH^low^ cells, measurements were made according to manufacturer’s instructions. Briefly, plates were removed from the incubator and allowed to equilibrate at room temperature for 20 min, and equal volume of CellTiter-Glo Luminescent Cell Viability Assay reagent was added directly to the wells. Plates were incubated at room temperature for 30 min on a shaker and luminescence was measured on an Envision reader (PerkinElmer; 570 nm).

### Tissue micro-array construction

Tissue microarrays (TMA) were constructed as previously described to represent endometrial cancer progression^15^. Archival formalin-fixed, paraffin embedded endometrial tissue samples were obtained with UCLA Institutional Review Board (IRB) approval from the David Geffen School of Medicine at UCLA, and the studies conducted in the laboratory were performed under approved guidelines. The IRB waived the need for informed consent as this was a retrospective analysis using de-identified samples. The study cohort consisted of 226 randomly selected patients who underwent endometrial sampling through a variety of biopsy, curettage or resection procedures from 1982 to 2002. Each histologic sample was represented by at least three 1 mm cores that were taken from donor paraffin embedded tissue blocks.

Of the 226 patients, 207 individuals had multiple samples representing those who did or did not develop endometrial cancer over the 20 year time period (“metachronous”). The array contained 1879 cores representing the following histologies: (1) benign endometrium (n = 231); (2) simple hyperplasia and complex hyperplasia (n = 141); (3) simple and complex atypical hyperplasia (n = 54); and (4) primary endometrial adenocarcinoma (n = 109). Atrophic, weakly proliferative, proliferative, secretory, disordered proliferative, progestational effects due to hormone therapy, and polypoid endometrium were all grouped as “benign” endometrium. Endometrial hyperplasia was classified based on glandular complexity and nuclear atypia with both simple and complex grouped as “hyperplasia” or “atypical hyperplasia”. All endometrial tumors were staged according to the TNM staging system endorsed by the American Joint Committee on Cancer (AJCC) and the International Union Against Cancer (UICC) and limited to endometrioid types^16^. Representative hematoxylin and eosin images are provided in Fig. S1. Low grade adenocarcinomas were identified based on architectural evidence of stromal invasion, usually in the form of stromal disappearance, desmoplasia, necrosis, or a combination of these findings between adjacent glands. Type I endometrial cancer variants such as ciliated, secretory, papillary (villoglandular), adenoacanthoma, and adenosquamous were included. For this particular analysis, 453 samples from 158 patients contained adequate ALDH1 immunostaining information for evaluation. Of these, 33 patients had metachronous samples with disease progression to cancer. Table 1 summarizes clinical variables and patient groups as well as ALDH1 expression in each subgroup.

**Table 1:** Clinical variables and patient groups stratified based on cytoplasmic ALDH1 expression.

	All patients	ALDH1 negative (% of total)	ALDH1 positive (% of total)	*p* value
**Total**	158	105 (66%)	53 (34%)	
**BMI (kg/m**^**2**^**)**				
Median (range)	27 (16–57)	27 (16–56)	28 (20–57)	0.13^a^
Mean	29	28	30	
BMI < 30	83 (60%)	56 (40%)	27 (19%)	0.72^b^
BMI ≥ 30	56 (40%)	36 (26%)	20 (14%)	
**Ethnicity**				
Others	28 (19%)	17 (11%)	11 (7%)	0.50^b^
Caucasian	122 (81%)	84 (56%)	38 (25%)	
**Age of 1st biopsy (years)**				
Median (range)	46 (24–83)	46 (24–74)	47 (29–83)	0.87^a^
Mean	47	47	47	
**Age of EC diagnosis (years)**				
Median (range)	56 (30–86)	60 (30–79)	55 (35–86)	0.31^a^
Mean	57	58	55	
Number of women	41	23	18	
**Follow-up time (months)**				
Mean	64	72	49	0.01^a^
Median (range)	54 (0–249)	55 (0–249)	45 (0–176)	
**Smoking history**				
Yes	50 (35%)	32 (22%)	18 (13%)	0.86^b^
No	94 (65%)	62 (43%)	32 (22%)	
**Gravidity**				
Median (range)	2 (0–8)	2 (0–8)	2 (0–6)	0.80^b^
**Parity**				
Median (range)	1 (0–6)	1 (0–6)	1 (0–4)	0.81^b^
0–1	78 (53%)	48 (33%)	30 (21%)	
≥ 2	68 (47%)	47 (32%)	21 (14%)	
**Menopause (years)**				
Median (range)	50 (31–59)	50 (31–59)	50 (34–56)	0.69^a^
Mean	49	49	49	
< 48 years	38 (27%)	24 (17%)	14 (30%)	
48–52 years	71 (51%)	49 (35%)	22 (16%)	
> 52 years	31 (22%)	21 (15%)	10 (7%)	
**Diabetes mellitus**				
No	113 (80%)	75 (53%)	38 (27%)	0.67^b^
Yes	29 (20%)	18 (13%)	11 (8%)	
**Estrogen therapy history**				
Yes	82 (53%)	57 (37%)	25 (16%)	0.61^b^
No	73 (47%)	47 (30%)	26 (17%)	
**Progesterone therapy history**				
Yes	109 (70%)	73 (47%)	36 (23%)	1^b^
No	46 (30%)	31 (20%)	15 (10%)	

^a^Mann–Whitney.

^b^Fisher's Exact.

All the cases were reviewed using the World Health Organization histological criteria for the diagnosis of endometrial carcinoma and hyperplasia^2^. We evaluated the hematoxylin and eosin stained slides for gland-to-stroma ratio (glandular crowding), architectural abnormalities (gland confluency, cribriforming, and papillary architecture). We also evaluated the sections for cytological atypia which includes nucleus-to-cytoplasm ratio, presence and prominence of nucleoli, nuclear chromatin quality, and mitotic activity by light microscopy. If there was an increase in gland-to stroma ratio or architectural atypia with or without cytological atypia, those cases were considered as no response to therapy^2^. Distinction of well differentiated endometrioid carcinoma from atypical hyperplasia/endometrial intraepithelial neoplasia was based on the presence of stromal invasion, altered endometrial stroma or a papillary architecture as per WHO guidelines^2^.

### Immunohistochemistry

Five-micrometer thick TMA sections were de-paraffinized in three washes of xylene and then rehydrated in serial dilutions of ethanol. Antigen retrieval was performed by placing the slides in a container of 0.1 mol/L citrate, pH 6.0, at 95 °C for 20 min. ALDH1 expression was detected using clone 44/ALDH (1:100; cat #611194, BD Biosciences, San Jose, CA, USA). Staining was visualized using DAKO EnVision + System, HRP (Agilent, Santa Clara, CA). Counterstaining was performed with hematoxylin. Slides were then placed in distilled water, dehydrated and mounted. An isotype control (MAB002, R&D Systems) was used for the negative control slides.

Results were analyzed by a pathologist (Y.E.) who performed scoring of the samples by rating the intensity from 0 to 3 (0 = below the level of detection, 1 = weak, 2 = moderate, 3 = strong) and percentage of cells staining at each intensity. A histologic score (H-score) was calculated for each sample by multiplying the percentage of positive cells by the intensity score. For cytoplasmic and nuclear expression, positive ALDH1 expression was defined as an H-score being larger than 0. For the purpose of reproducibility, two pathologists including one gynecological pathologist (N.A.M) and a general surgical pathologist (Y.E), both of who was blinded to the clinical data, have reviewed each slide and scored the morphology and location of ALDH1 + cells on each slide.

### Statistics

Statistical analyses were performed using R (http://www.R-project.org) including the 'survival' and 'survminer' packages. Pooling criteria were as previously described^15^. To examine differences in ALDH1 expression between samples, the Kruskal–Wallis test, Mann–Whitney U test and Spearman correlation were employed. The Kruskal–Wallis test was used to examine differences in ALDH1 expression in relation to the development of cancer. A mean pooled H-score for ALDH1 was calculated across the three cores used in each histologic sample. Since intensity of staining did not seem to affect the outcomes of the results, we used percentage of positivity alone for our calculations. The dependence between categorical variables was tested using the Fisher’s exact test. Estimation curves for probability of cancer-free survival were generated using the Kaplan–Meier method, and comparisons made using the log-rank test. Hazard ratios and prognostic significance of ALDH1 expression were estimated using the Cox proportional hazards model. For barplots, data is presented as the mean expression ± standard error of the mean (SEM). For all results, *p* < 0.05 was considered significant.

## Results

### ALDH expression in endometrial cancer

**Figure 1:**
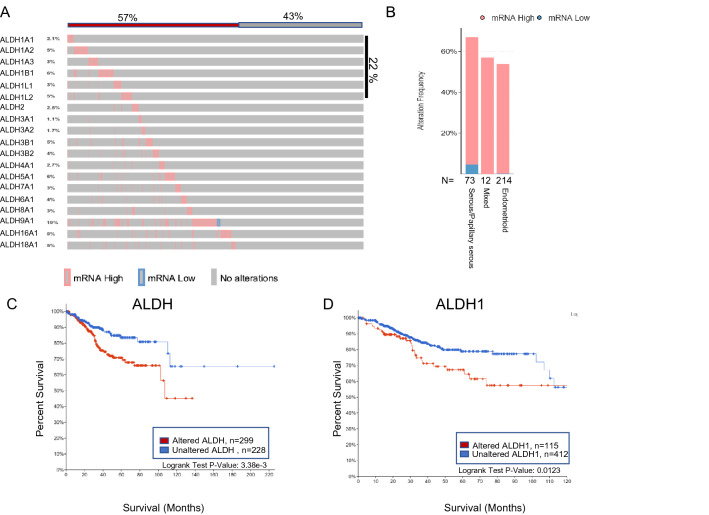
(**A**) ALDH expression was evaluated in the PanCancer Atlas. An OncoPrint depicts the results and shows the graphical summary of mRNA alternations in all 19 ALDH isoforms across a set of 527 endometrial tumor samples (TCGA, PanCancer Atlas). (**B**) Distribution of ALDH isoforms in serous/papillary, mixed or endometrioid endometrial cancer. (**C**) Kaplan–Meier plot showing overall survival of Endometrial Carcinoma patients from the TCGA database with altered (red, number of patients, *n* = 299) or unaltered ALDH expression (blue, number of patients, *n* = 228). The Two-sided log-rank *P* value is displayed. (**C**) Kaplan–Meier plot showing overall survival of Endometrial Carcinoma patients from the TCGA database with altered (red; number of patients, *n* = 115) or unaltered ALDH1 expression (blue, number of patients, *n* = 412). A two-sided log-rank *P* value is displayed.

High ALDH activity has been associated with self-renewal in a variety of normal and tumor tissues including the prostate, breast, lung, colon, cervix, and ovary^17^, but little is known about its expression in the endometrium. As a starting point, all 18 isoforms were queried using the The Cancer Genome Atlas (TCGA) PanCancer Atlas in 527 patients with uterine Corpus Endometrial Carcinoma^18^. Within all endometrial cancer subtypes, ALDH was present in 57% of patients, with ALDH1 representing the cumulative dominant isoform (Fig. 1A). ALDH isoforms were present in all subtypes of endometrial cancer including serous, papillary, and endometrioid (Fig. 1B). Analysis revealed upregulation of ALDH mRNA in 62.4% of serous/papillary tumors, 57.1% of mixed, and 53.7% of endometrioid endometrial cancers. To understand the significance of this, we evaluated all ALDH isoforms as well as only ALDH1 for potential correlation with survival. To perform this analysis, we utilized The Cancer Genome Atlas (TCGA) and found a significant correlation between overall survival and high expression of the ALDH gene signatures (Fig. 1C). Within the TCGA endometrial cancer cohort, ALDH1 also correlated with survival (Fig. 1D).

### The ALDH^high^ subpopulation of endometrial cancer cells have CSC properties

**Figure 2:**
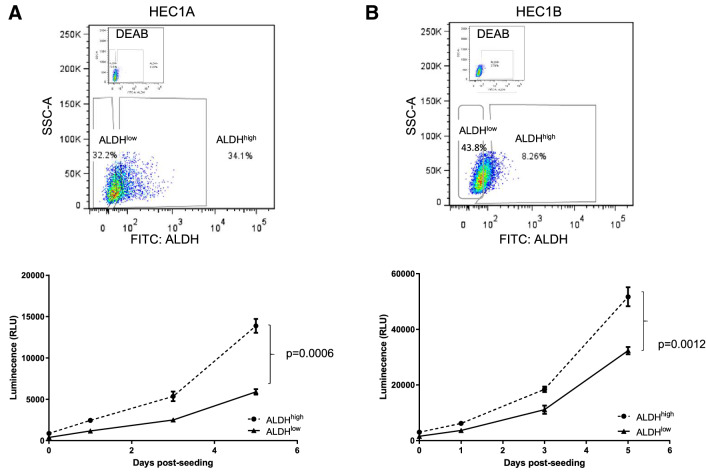
ALDH^high^ cells show enhanced proliferation. (**A**) Top, Flow diagram showing the gating of ALDH^high^ and ALDH^low^ HEC1A cells. Bottom, The proliferation of sorted populations was evaluated over 5 days. Each sample was analyzed in triplicate with data shown as the mean ± SEM. The results shown are representative of three experiments. Two way ANOVA, *p* = 0.0006. (**B**) ALDH^high^ and ALDH^low^ HEC1B cells were sorted as above (top panel), with cellular proliferation analyzed over 5 days (bottom panel). Each sample was analyzed in triplicate with data shown as the mean ± SEM. The results shown are representative of three experiments. Two-way ANOVA, *p* = 0.0012.

To next verify that ALDHhigh versus ALDHlow cells show biological differences, we determined whether ALDH activity could enrich for cells with a higher proliferative capacity in vitro. HEC1A and HEC1B endometrial cancer cell lines were sorted by FACS and designated as ALDH^high^ or ALDH^low^ cell population. To compare the biological behaviour of these two sorted subpopulations, we evaluated their growth curves in vitro. Compared to ALDH^low^ cells, ALDH^high^ cells grew faster over a 5 day incubation period (Fig. 2).

### Clinical characteristics

While ALDH activity has been implicated with cancer stem properties, little is known about its role in tumor progression. To address this gap, endometrial tissue from 158 patients was analyzed for ALDH1 expression, with clinical characteristics listed in Table 1. The median BMI was 27 (range 16–57), with 40% of the patients meeting the CDC criteria for obesity (BMI > 30). At some time, 20% of patients developed diabetes mellitus. Caucasians represented the major ethnic group examined (81%) while Hispanic/Latinas and African Americans comprised 8% and 5% of the array cohort respectively. 35% of women smoked. In this cohort of women, the median age for cessation of menstruation occurred at 50 year old (range 31–59). The first biopsy occurred at 46 years old (median age; range 24–83).

### ALDH1 expression in normal endometrium

**Figure 3:**
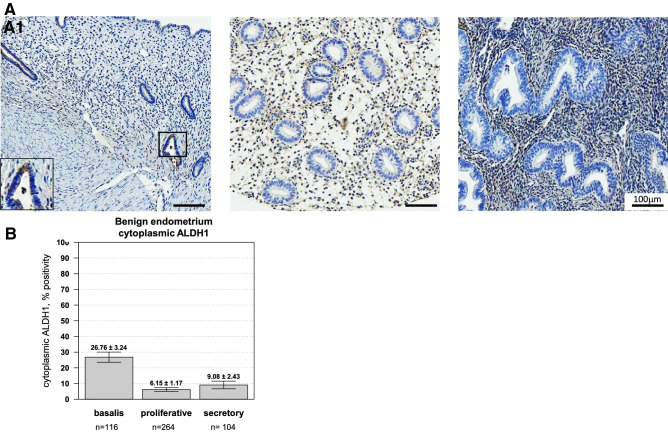
ALDH1 is expressed within the basal layer of the endometrium. (**A**) Representative cores representing the stratum basalis (**A1**) compared to proliferative (**A2**) or secretory (**A3**) endometrium. All images are 100X. The percentage of glands with positive expression were scored. In benign endometrial tissue, (**B**) Cytoplasmic ALDH1 expression was observed in endometrial glands of the stratum basalis (26.76 ± 3.24, n = 116 cores) compared to both proliferative and secretory endometrium (Kruskal–Wallis *p* value = 1.145e−14). Significantly lower expression of ALDH1 was observed in the proliferative phase (6.15 ± 1.17, n = 264 cores) and in the secretory phase (9.08 ± 2.43, n = 104).

Initially, we evaluated the cytoplasmic expression of ALDH1 in benign endometrium (Fig. 3). Expression within the stratum basalis was scored independently from the functional layer, with representative images presented in Fig. 3A. In the stratum basalis, weak to moderate cytoplasmic expression occurred in 26.8 ± 3.2% of endometrial stromal cells while benign proliferative and secretory glands showed negligible levels (6.2 ± 1.2 and 9.1 ± 2.4%, respectively) of ALDH1 expression (Fig. 3B; Kruskal–Wallis, *p* value = 1.1e−14).

Several papers have suggested that outside of the cytoplasm, ALDH1 expression can be present in the nucleus^19, 20^, and thus, this site was independently scored. Very low levels of nuclear staining were observed, with generally less than 1% of cores showing any expression (Fig. S2A).

### ALDH1 expression in glandular epithelium predicts malignant progression

**Figure 4:**
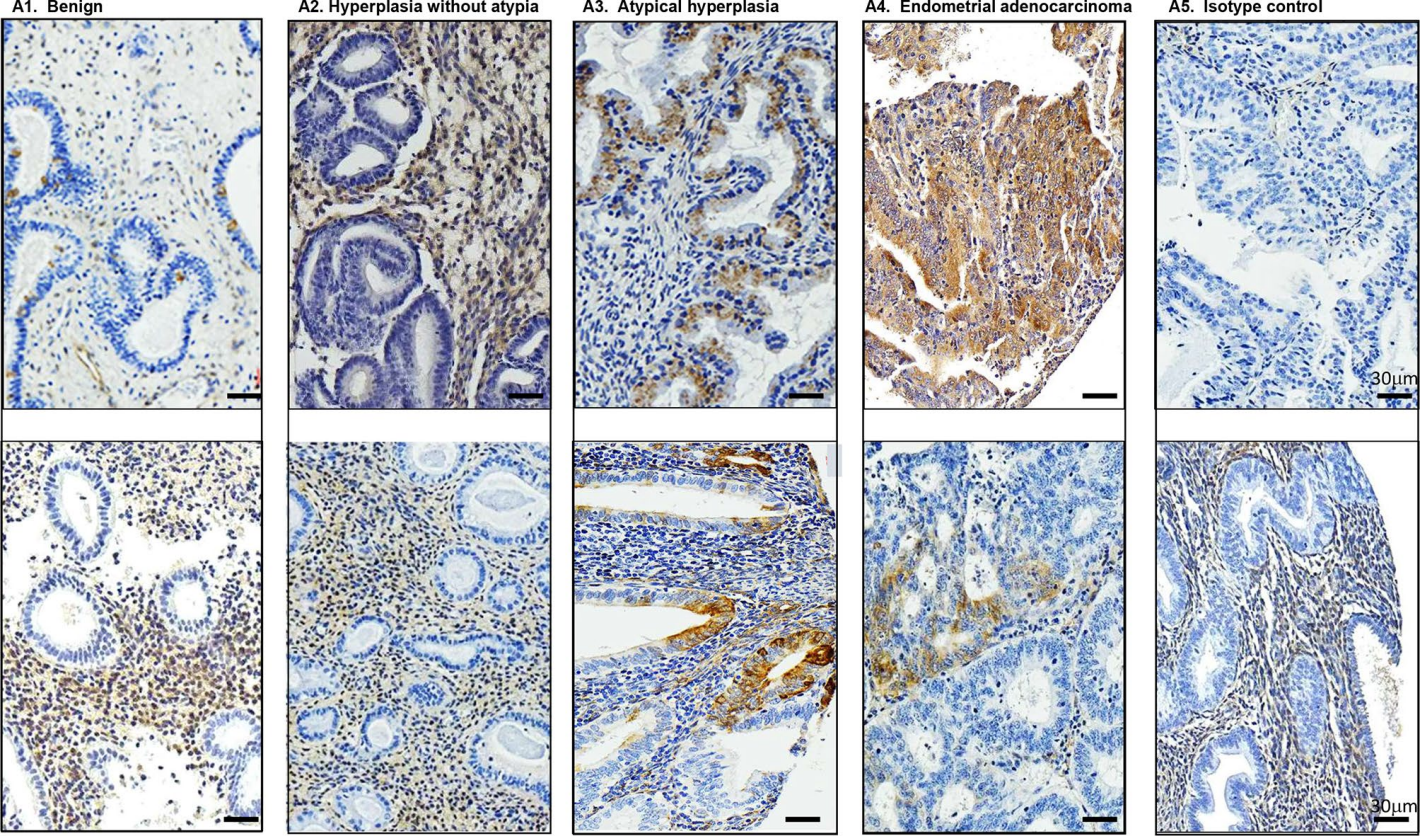
ALDH1 is expressed in premalignant endometrium. Immunohistochemical staining from representative cores of two patients per histology is shown. Within the endometrium, ALDH1 predominantly displayed a cytoplasmic distribution in glandular epithelium with some expression observed in the stroma. Magnification = 100X. Scale bar = 30 µm.

**Figure 5:**
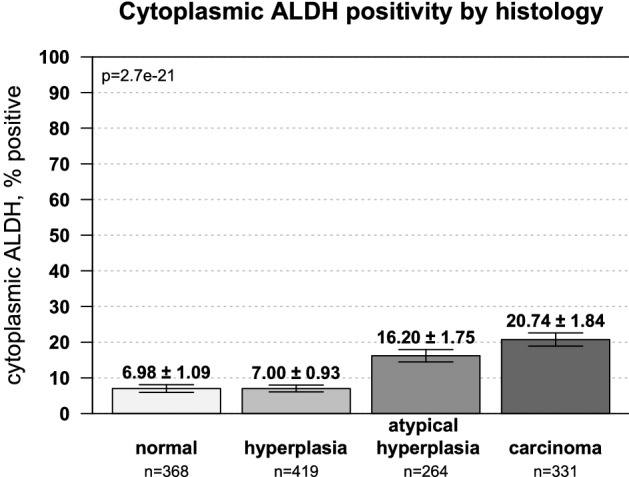
Epithelial ALDH1 expression increases occurs during malignant progression. The percentage of patients with epithelial ALDH1 expression showed a step-wise increase from endometrial hyperplasia to atypia to endometrial cancer. Spearman correlation for histologic progression was rho = 0.25 and *p* = 2.7e−21.

ALDH1 expression was next evaluated for each spot on the TMA and analyzed relative to each histology during malignant progression. Figure 4 illustrates representative images of immunohistochemical staining across histologic groups. Within the epithelia, benign endometrium and hyperplasia showed similar, very low levels of ALDH1 expression. However, progression from hyperplasia to endometrial cancer revealed a step-wise augmentation in cytoplasmic ALDH1 expression (Fig. 5A; Spearman correlation, rho = 0.25, *p* = 2.7e−21). The mean cytoplasmic positivity rose more than two-fold in atypia (from 7.0% ± 0.9 to 16.2% ± 1.8%) and further increased in endometrial carcinoma (20.7% ± 1.8%). Nuclear staining for ALDH1 remained very low in all histologies and showed only weak correlation with progression of hyperplasia to malignancy (Fig. S2B; rho = 0.06, *p* = 0.03).

Interestingly, ALDH1 positivity at the time of first biopsy did not correlate with many variables commonly associated with endometrial cancer development including age or BMI, and its expression did not associate with an early onset of menopause (Table 1). In the patient cohort examined, 83% of patients were on hormone replacement therapy, with 45% on estrogen and progesterone at some point during their follow-up. When examining each hormone independently, no correlation between ALDH1 positivity and the use of estrogen and/or progesterone were observed. However, ALDH1 expression did correlate with follow-up time or the number of months from the date of the first informative surgical pathology report to the date of the last surgical intervention. ALDH1 positive tumors correlated with a shorter follow-up time with patients requiring surgical intervention 23 months earlier than those with negative tumors (Table 1).

**Figure 6:**
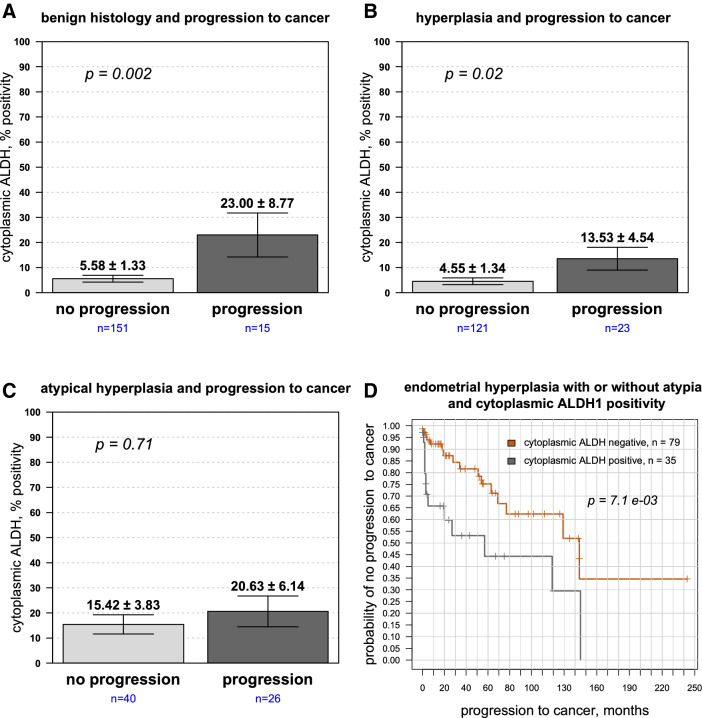
ALDH1 cytoplasmic expression in epithelia is upregulated in patients who ultimately develop endometrial cancer. ALDH1 expression was evaluated in epithelia from patients with (**A**) histologically normal endometrium, (**B**) hyperplasia without atypia and (**C**) atypical hyperplasia, with expression dichotomized into those who developed or did not develop cancer. Epithelial ALDH1 expression predicted increased likelihood of progression to developing cancer in patients with benign endometrium (*p* = 0.002) and hyperplasia (*p* = 0.02), but did not reach significance in atypical hyperplasia. (**D**) Kaplan–Meier plot showing time to progression to cancer for cytoplasmic ALDH1 expression in endometrial hyperplasia with or without atypia: the difference in median time to cancer diagnosis 145 months when ALDH1 negative vs 56 months when ALDH1 was positive, HR = 2.66, *p* = 7.1e−3.

The results thus far suggested that cytoplasmic ALDH1 positively correlated with malignant progression. Within each histological group, however, there was heterogeneity in ALDH1 expression with some individuals showing higher ALDH1 levels than others. We therefore examined whether ALDH1 provided information regarding future tumor development. First, we dichotomized ALDH1 expression from patients in each histologic group into those who developed or did not develop cancer. In normal tissue, ALDH1 largely resides in epithelia found in the stratum basalis. During premalignant progression, an increase occurred in cytoplasmic ALDH1 expression in patients who went on to develop cancer compared to those who did not (Fig. 6A–C). In all cases, amplified ALDH1 trended in patients where disease progressed. Histologically benign tissue from patients who ultimately went on to develop cancer showed statistically significant increased cytoplasmic ALDH1 positivity compared to those who did not (Fig. 6A; 5.6 ± 1.3% compared to 23 ± 8.8%, *p* = 0.002). Similarly, comparing patients with endometrial hyperplasia but without atypia, higher ALDH1 positively was observed in women who went on to develop cancer compared to those who did not (Fig. 6B; 13.5 ± 4.5% compared to 4.6 ± 1.3%, *p* = 0.02) Higher mean cytoplasmic ALDH1 expression in patients who developed endometrial cancer when initially presenting with atypical hyperplasia was also observed, but this trend was not statistically significant (Fig. 6C; *p* = 0.71). Nonetheless, the results collectively suggest that positive ALDH1 in the epithelium may enhance the malignant potential of hyperplastic endometrium.

We further analyzed this data using the Cox proportional hazards model with cytoplasmic ALDH1 as a continuous variable predictor (Table 2). In patients with hyperplasia or atypical hyperplasia, higher ALDH1 epithelial expression was associated with increased risk for progression to carcinoma (*p* = 1.4e−3). A Kaplan–Meier plot (Fig. 6D) for patients with endometrial hyperplasia with or without atypia showed a shorter time interval for progression to cancer in those with ALDH1 positive cells than those that were completely negative (median time 57 months vs 144 months, hazard ratio (HR) = 2.66, 95% CI 1.27–5.58; *p* = 7.1e−03).

**Table 2:** Univariate Cox proportional hazards analysis for ALDH1 as a predictor of development of carcinoma in patients with hyperplasia or atypical hyperplasia.

Cellular compartment	Hazard ratio (95% confidence interval)	*p* value
Epithelial expression	1.022 (1.007–1.036)	1.4e−03
Stromal expression	0.985 (0.974–0.996)	6.91e−03

### ALDH1 stromal staining

**Figure 7:**
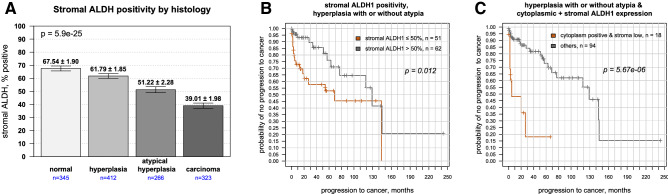
Stromal ALDH1 expression in premalignant lesions predict future tumor development. (**A**) Stromal ALDH1 expression showed a step-wise decrease from normal endometrium to endometrial hyperplasia to atypical hyperplasia to endometrial cancer (Spearman rho = − 0.28, *p* = 5.9e−25). (**B**) Stromal ALDH1 expression reduced the likelihood of patients to develop cancer. Kaplan–Meier survival plot showing higher stromal ALDH1 expression (> 50% of cells) versus lower expression in hyperplastic lesions with or without atypia: difference in median time to cancer diagnosis 129 months vs 69 months, HR = 0.39, *p* = 0.012. E) Patients with ALDH1 positivity in the epithelium and low immunostaining in stroma (< 50%) also showed shorter times to cancer diagnosis compared to others (median time interval 6 months vs 129 months, *p* = 5.67e−06).

An emerging issue in tumor progression centers on the interactions between cancer cells and the microenvironment, and several studies have suggested a role for stroma in inhibiting tumor growth^21, 22^. As prominent stromal staining occurred in several cores, its relative expression was assessed during stages of malignant progression. ALDH1 levels were highest in benign stroma and hyperplasia without atypia, then steadily decreased during progression to malignancy, with the lowest levels observed in endometrial cancer (Fig. 7A, rho = − 0.28, *p* = 5.9e−25). When we next examined patients with endometrial hyperplasia, stromal expression of ALDH1 did not predict patients who developed cancer. However, for patients with atypical hyperplasia, lower levels were significantly associated with increased risk of progression to cancer (*p* = 0.002). Mean stromal positivity was 58.2 ± 5.9% in those who did not develop cancer and 30.1 ± 5.8% in those who did.

Applying the continuous Cox proportional hazard model, higher percentage of stromal cells positive for ALDH1 conferred protection, with a hazard ratio of 0.98 (95% CI 0.974–0.996) and *p* = 6.9e−3 (Table 2). Stromal ALDH1 expression was next assessed using Kaplan–Meier estimates for disease progression. Patients with higher stromal expression showed a longer median cancer free interval compared to patients with lower levels (130 vs. 70 months respectively; *p* = 0.012) (Fig. 7B). When the results were merged, any glandular cytoplasmic positivity along with lower levels of stromal staining (cut point again defined by 50% expression level for stroma) suggested significantly poorer outcomes (Fig. 7C). Bivariate analysis showed a difference in median cancer free interval of 130 months vs 6 months (*p* = 5.67e−06). Collectively, our results reveal an independent effect of ALDH1 within the stroma and epithelium.

## Discussion

Given the recent interest in cancer stem cells as therapeutic targets for decreasing potential of cancer metastasis and relapse, it is important to investigate the expression of cancer stem cell markers in pre-cancerous tissues and to understand the significance of the expression of these markers within a clinical context of predicting outcomes. High cytoplasmic ALDH1 expression predicts poor prognosis and/or increased tumor aggressiveness in various other cancer types^23–25^, and TCGA analysis suggests that ALDH isoforms correlate with poor survival and enhanced proliferation in this study on patients with endometrial cancer. However, few studies to date have used ALDH1 to not just predict cancer aggressiveness but to even predict the development of cancer from potentially pre-neoplastic tissue. These studies, generally limited to the development of oral squamous cell carcinoma^26, 27^, have shown ALDH1 expression associated with a three-fold increased risk for the development of cancer^25^. Our study is the first to our knowledge to demonstrate the utility of ALDH1 to predict endometrial cancer development.

In addition, our study is the first to demonstrate that in normal endometrial glands cytoplasmic ALDH1 expression largely resides within the basalis. This distinction may have important pathophysiologic implication as others have shown that high ALDH activity has been associated with stem and progenitor cells in various tissues^28, 29^. ALDH1, in particular, is highly expressed in hematopoietic progenitors, in intestinal crypt cells, as well as in normal mammary stem cells^17, 30, 31^. In addition, high expression has been observed in hematopoietic stem cells (HSCs) where its effects influence retinoid metabolism^32^. Functionally, given that the ALDH superfamily of enzymes have been shown to catalyze the formation of retinoic acid and to be critical for the detoxification of endogenous and exogenous aldehydes^17^, we hypothesize that ALDH1 + cells within the basalis may identify the endogenous stem cell population of the endometrial glands.

Notably, epithelial cell staining of ALDH1 did not correlate with stromal expression. Instead, ALDH1 positive stromal staining was highest in benign endometrium and lowest in endometrial cancer. While its expression in the stroma has been well documented in multiple cancer types, the clinical implications of its expression have not been well established. Within the tumor microenvironment, multiple cell types including fibroblasts, endothelial cells, and immune cells shape epithelial cell maintenance and regeneration^33^, and several studies have shown that stromal signals can regulate epithelial cell growth and progression in multiple tumor types, including those of the breast, pancreas, colon and prostate^33–35^. Our results suggest, similar to recent studies in the breast^21, 36^, that ALDH1 positive stroma offers a potential protective effect in the endometrium.

Given these findings and its putative roles in cell differentiation, ALDH1 positivity within endometrial epithelia seems to be a biologically important marker of cancer stem cell activity. We have demonstrated that ALDH1 expression can predict cancer development, in addition to predicting patient survival as was demonstrated by others^37, 38^. Though it remains unknown whether ALDH1 plays a causative role in the transition of hyperplasia/atypia to malignancy, it is clear that the reported actions of ALDH1 may contribute to several of the behavioral properties of CSCs and potentially predict responsiveness to therapy. For example, ALDH enzymes scavenge reactive oxygen species, protecting these cells from apoptosis, perhaps contributing to aggressive CSC behavior and resistance from targeted and chemical therapies^9, 39^.

In conclusion, we found in our study that while ALDH1 expression in endometrial epithelia predicts progression from hyperplasia and atypia to cancer, within the stroma it offers a protective effect. Additional studies will be needed to determine if there is any cross-talk between ALDH enzymes within these two compartments or if each is regulated independently.

## Supplementary Information

Supplementary Figures.
