# Supplementary material for: ALDH1 expression predicts progression of premalignant lesions to cancer in Type I endometrial carcinomas
Source: Sci Rep. 2021 Jun 7;11:11949. doi: 10.1038/s41598-021-90570-3 (PMC8184965; doi:10.1038/s41598-021-90570-3)
Supplement: Supplementary file 1 — Supplementary Figures. [file 41598_2021_90570_MOESM1_ESM.pdf]

Supplementary Figure 1

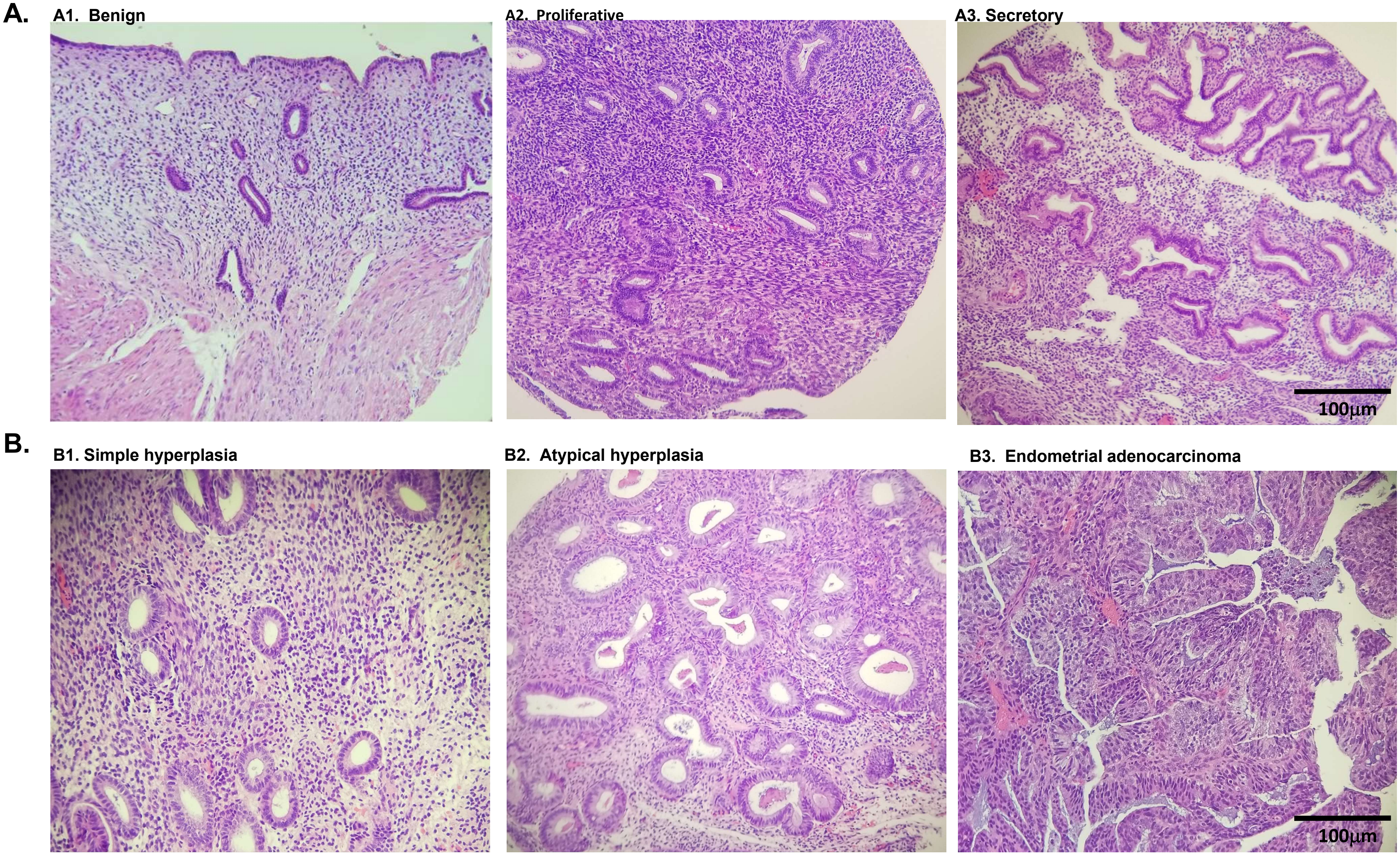

Fig S1.Progression to endometrial cancer histology. Hemotoxylin and eosin staining illustrates benign, premalignant and malignant endometrium. Magnification=200X.

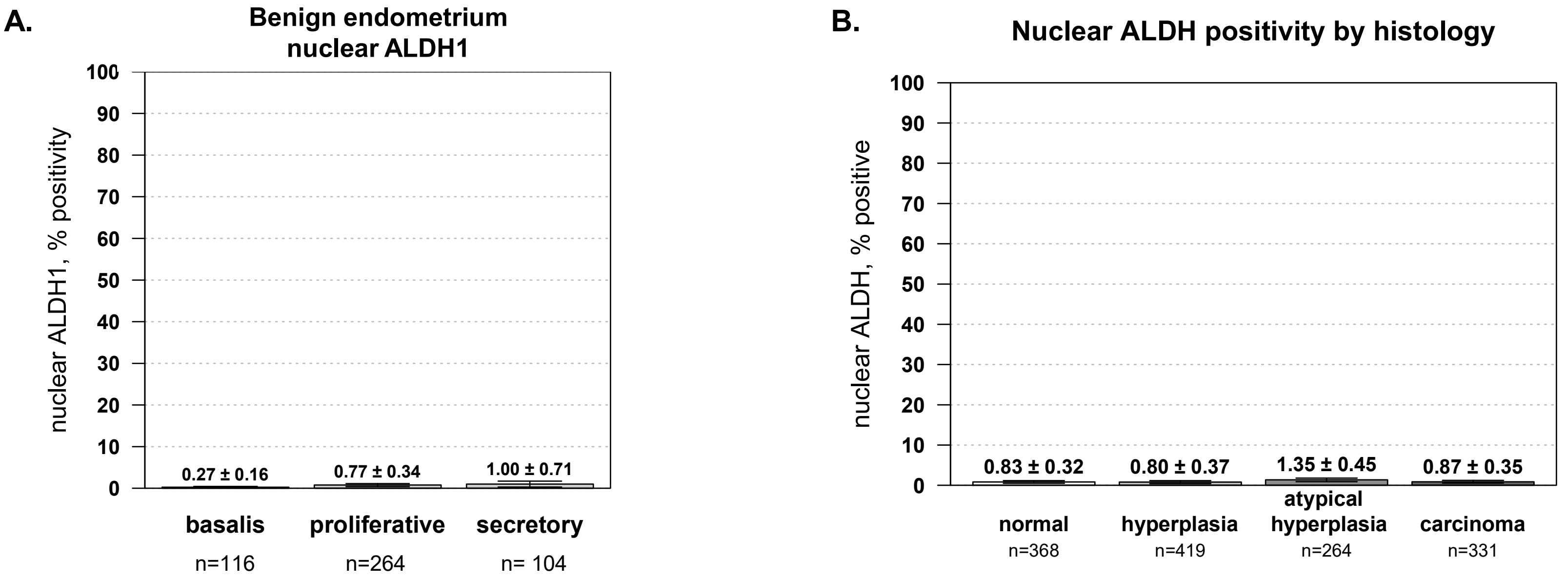

A.
Benign endometrium
nuclear ALDH1
nuclear ALDH1, % positivity
100
90
80
70
60
50
40
30
20
10
0
0.27 ± 0.16
0.77 ± 0.34
1.00 ± 0.71
basalis
proliferative
secretory
n=116
n=264
n= 104
B.
Nuclear ALDH positivity by histology
nuclear ALDH, % positive
0.83 ± 0.32
0.80 ± 0.37
1.35 ± 0.45
0.87 ± 0.35
normal
hyperplasia
atypical hyperplasia
carcinoma
n=368
n=419
n=264
n=331

Fig S2. Nuclear expression of ALDH1. Low levels of ALDH1 were observed in benign or premalignant nuclei.
